# New insight of the pathogenesis in osteoarthritis: the intricate interplay of ferroptosis and autophagy mediated by mitophagy/chaperone-mediated autophagy

**DOI:** 10.3389/fcell.2023.1297024

**Published:** 2023-12-08

**Authors:** Fangyu An, Jie Zhang, Peng Gao, Zhipan Xiao, Weirong Chang, Jiayi Song, Yujie Wang, Haizhen Ma, Rui Zhang, Zhendong Chen, Chunlu Yan

**Affiliations:** ^1^ Teaching Experiment Training Center, Gansu University of Chinese Medicine, Lanzhou, China; ^2^ School of Basic Medicine, Gansu University of Chinese Medicine, Lanzhou, China; ^3^ School of Traditional Chinese and Western Medicine, Gansu University of Chinese Medicine, Lanzhou, China; ^4^ Teaching Department of Medicine, Gansu University of Chinese Medicine, Lanzhou, China

**Keywords:** osteoarthritis, ferroptosis, mitophagy, chaperone-mediated autophagy, reactive oxygen species, adenosine monophosphate (AMP)-activated protein kinase (AMPK), hypoxia-inducible factors

## Abstract

Ferroptosis, characterized by iron accumulation and lipid peroxidation, is a form of iron-driven cell death. Mitophagy is a type of selective autophagy, where degradation of damaged mitochondria is the key mechanism for maintaining mitochondrial homeostasis. Additionally, Chaperone-mediated autophagy (CMA) is a biological process that transports individual cytoplasmic proteins to lysosomes for degradation through companion molecules such as heat shock proteins. Research has demonstrated the involvement of ferroptosis, mitophagy, and CMA in the pathological progression of Osteoarthritis (OA). Furthermore, research has indicated a significant correlation between alterations in the expression of reactive oxygen species (ROS), adenosine monophosphate (AMP)-activated protein kinase (AMPK), and hypoxia-inducible factors (HIFs) and the occurrence of OA, particularly in relation to ferroptosis and mitophagy. In light of these findings, our study aims to assess the regulatory functions of ferroptosis and mitophagy/CMA in the pathogenesis of OA. Additionally, we propose a mechanism of crosstalk between ferroptosis and mitophagy, while also examining potential pharmacological interventions for targeted therapy in OA. Ultimately, our research endeavors to offer novel insights and directions for the prevention and treatment of OA.

## 1 Introduction

Osteoarthritis (OA) is a common degenerative joint disease in the elderly, with joint pain, decreased mobility, and stiffness as the main clinical manifestations. The pathological features include synovitis, chondrocyte apoptosis, chondrocyte extracellular matrix (ECM) degradation, subchondral osteosclerosis, and osteophyte formation (Martel-Pelletier et al., 2016), with high incidence and disability rates (Glyn-Jones et al., 2015) mations based on epidemiological data shows that, globally, approximately 240 million people suffer from symptomatic OA, of which 10% are men and 18% are women above 60 years old (Allen et al., 2022). The prevalence rate of OA in people over 75 years old is between 70% and 90% (Lane and Corr, 2017). A population-based epidemiological study shows that the prevalence of OA will most likely increase yearly in the next 10 years (Turkiewicz et al., 2014). This will greatly increase economic burdens and societal pressures (Peat and Thomas, 2021) and contribute to disability levels in the elderly (Kloppenburg and Berenbaum, 2020). Therefore, there is an urgent need to treat OA.

Articular cartilage primarily consists of chondrocytes and ECM, which predominantly comprises organic components such as water, collagen Ⅱ (Col2), aggrecans, and other proteoglycans. Chondrocytes are the sole cellular component found in articular cartilage, and alterations in chondrocytes serve as the fundamental etiology of OA disease (Rim et al., 2020). Research has provided evidence that proinflammatory cytokines, including tumor necrosis factor-α (TNF-α), interleukin-1β (IL-1β), prostaglandins (PG), and nitric oxide synthase (NOS), have the ability to stimulate chondrocytes to secrete catabolic factors such as A disintegrin-like and metalloprotease domain with thrombospondin type 1 repeats (ADAMTS) metalloproteinase, matrix metalloproteinases (MMPs), cyclooxygenase-2 (COX-2), and nitric oxide synthase (NOS). These inflammatory factors also inhibit the synthesis of Col2, proteoglycans, and other synthetic metabolic factors, thereby promoting a greater occurrence of chondrocyte catabolic reactions compared to synthetic metabolic reactions, thus having a destructive effect on the articular cartilage (Hosseinzadeh et al., 2016; Robinson et al., 2016). Currently, there is a prevailing belief in the academic community that chronic inflammation, oxidative stress, ferroptosis, and mitophagy damage can stimulate chondrocytes to overproduce ADAMTS, MMPs, COX-2, NOS, and other catabolic factors. This excessive production ultimately results in the deterioration of chondrocytes and ECM, thereby hastening the degeneration of articular cartilage in OA (Hui et al., 2016; Wang et al., 2020a; Hodgkinson et al., 2022; Kawata et al., 2022). Among these factors, ferroptosis and mitophagy/CMA have garnered significant attention in the occurrence and progression of OA (Sun et al., 2021; Yang et al., 2022a; Lorenzo-Gómez et al., 2023). Ferroptosis primarily manifests through elevated reactive oxygen species (ROS), lipid peroxidation, iron overload, and glutathione (GSH) deprivation (Stockwell et al., 2017). Studies have demonstrated the presence of ferroptosis in chronic bone diseases (Yang et al., 2022b; Yan et al., 2022), including OA (Liu et al., 2022a; Miao et al., 2022). Mitophagy is a type of selective autophagy, where degradation of damaged mitochondria is the key mechanism for maintaining mitochondrial homeostasis (Palikaras et al., 2018). Recent findings suggest that the involvement of mitohagy/chaperone-mediated autophagy (CMA) in the pathogenesis of OA (Chen et al., 2021a; Lorenzo-Gómez et al., 2023). Impairment of mitophagy/CMA can result in excessive accumulation of ROS in chondrocytes, leading to oxidative stress-induced damage, chondrocyte apoptosis, and degradation of the ECM.

The accumulation of ROS can result in lipid peroxidation, leading to the induction of ferroptosis in chondrocytes and exacerbating OA. Conversely, moderate activation of autophagy can mitigate ROS production in degenerating articular cartilage cells, thereby alleviating OA. Consequently, enhancing the body’s antioxidant capacity and eliminating excessive ROS may prove to be an effective therapeutic approach for treating OA. Furthermore, the involvement of AMP-activated protein kinase (AMPK) and hypoxia-inducible factors (HIFs) in the pathogenesis and progression of OA, as well as their close association with ferroptosis and mitophagy, should also be considered (Hu et al., 2020; Li et al., 2020; Lu et al., 2021; Zhou et al., 2021; Jin et al., 2022; Guan et al., 2023; Xie et al., 2023). Additionally, there exists evidence indicating that the occurrence of ferroptosis is concomitant with heightened ferritin phagocytosis (Zhou et al., 2020). However, the precise regulatory mechanism of crosstalk between ferroptosis and mitophagy in the context of OA remains unclear. Consequently, it is imperative to comprehend the role of ferroptosis and mitophagy/CMA in the initiation and progression of OA. This article aims to elucidate the pathological mechanisms and potential crosstalk mechanisms underlying ferroptosis and mitophagy/CMA in the context of OA. Lastly, we underscore the potential transformative prospects of OA treatment.

## 2 Ferroptosis

Ferroptosis, proposed by Dixon in 2012, is a new type of non-apoptotic programmed cell death characterised by the iron-dependent accumulation of intracellular lipid ROS (Dixon et al., 2012). The morphological features include mitochondrial cristae rupture, reduction or disappearance, mitochondrial outer membrane destruction, and mitochondrial shrinkage (Stockwell et al., 2017; Gao et al., 2019a). The metabolic characteristics are iron overload, accumulation of ROS, and inactivation of the antioxidant enzyme glutathione peroxidase 4 (GPX4) (Seibt et al., 2019). Inhibition of GPX4 activity and reduction in the synthesis and release of GSH, can lead to accumulation of ROS lipid in membrane, thus inducing ferroptosis (Hu et al., 2021; Krümmel et al., 2021). The main pathways inducing ferroptosis are the antioxidant systemXc^−^/GSH/GPX4 regulatory axis, lipid peroxidation, and iron overload.

### 2.1 SystemXc^−^/GSH/GPX4 regulatory axis

**FIGURE 1:**
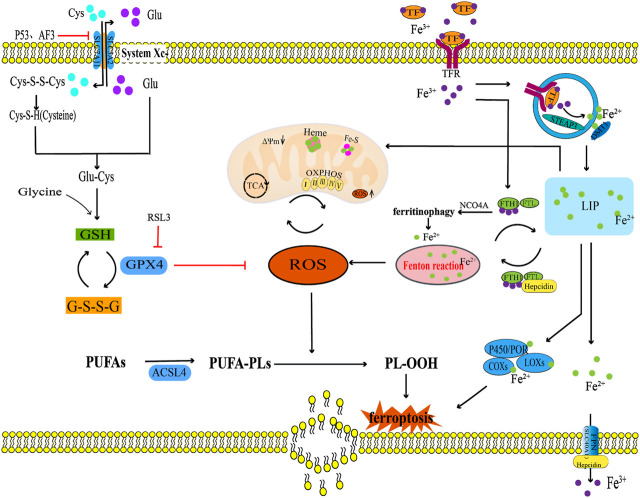
The ferroptosis regulation pathway. This figure summarizes the systemXc^−^/GSH/GPX4 regulatory axis, lipid peroxidation, and iron metabolism in the process of ferroptosis. The black arrow indicates activation and the red suppression arrow indicates inhibition.

The systemXc^−^/GSH/GPX4 regulatory axis in the body is considered the classical antioxidant system regulation axis of ferroptosis. The systemXc^−^ is an upstream molecule of the systemXc^−^/GSH/GPX4 regulatory axis, which is a sodium-independent reverse transporter embedded in the cell membrane. SystemXc^−^ comprises catalytic subunit xCT/member 11 of solute carrier family 7 (SLC7A11) and the regulatory subunit 4F2 (4F2hc)/member 2 of solute carrier family 3 (SLC3A2) (Gochenauer and Robinson, 2001). Among them, SLC7A11 is a 12-pass transmembrane protein light chain, with both the N-terminus and C-terminus located in the cytoplasm, responsible for primary transport activity and highly specific for cystine and glutamic acid. SLC3A2 is a single transmembrane protein heavy chain, with an N-terminus located inside the cell and a highly glycosylated C-terminus domain located outside the cell. SLC3A2 mainly acts as a chaperone protein and has a very important role in regulating the transport of light chain SLC7A11 to the plasma membrane (Sato et al., 1999). The main function of the systemXc^−^ is to transport intracellular glutamate and extracellular cystine in a 1:1 ratio (Bannai, 1986). Cystine entering cell is converted into cysteine (Cys) via a redox reaction, which then condenses with glutamic acid (Glu) and glycine (Gly) to form GSH (Conrad and Sato, 2012). The body promotes the reduction of oxidised GSH (GSSG) to reduced GSH through GPX4, thus protecting cells from peroxidation damage (Su et al., 2019). GPX4 is considered the most important antioxidant enzyme in mammals (Seiler et al., 2008). Research (Shao et al., 2022) has found that the tumor suppressor gene P53 can reduce the production of GSH and inhibit the activity of GPX4 by inhibiting the mRNA and protein expression of SLC7A11, thereby inducing ferroptosis. Another study (Wang et al., 2020b) also found that a common pressure sensor gene activating transcription factor 3 (ATF3) was obviously upregulated in erastin-induced ferroptosis of retinal pigment epithelium cells. ATF3, as a repressor of the systemXc^−^, futher downregulated the expression of SLC7A11 and reduced the production of GSH, thereby promoting ferroptosis. However, the overexpression of SLC7A11 can resist ferroptosis induced by erastin. It can be seen that the systemXc^−^ plays a key role in the regulation of ferroptosis. Futhermore, studies (Yang et al., 2014) have found that reduced GSH and GSSG are significantly increase the lipid ROS content in erastin-induced ferroptosis of HT-1080 fibrosarcoma cells. This indicates that the significant consumption of GSH/GSSG, as a major cell antioxidant system can induce ferroptosis. Jelinek et al. also found that in the ferroptosis models induced by RSL3 in neuron HT22 cells and mouse embryonic fibroblasts, inhibiting the activity of GPX4 can lead to lipid peroxidation, ROS accumulation, and mitochondrial dysfunction, which leads to ferroptosis (Jelinek et al., 2018). In summary, the systemXc^−^/GSH/GPX4 regulatory axis plays an important regulatory role in mediating ferroptosis, among which the systemXc^−^ may be a crucial initiating link in inducing ferroptosis (Figure 1).

### 2.2 Lipid peroxidation

Lipid peroxidation is one of the characteristics of ferroptosis that primarily involves the oxidative decomposition of polyunsaturated fatty acids (PUFAs) in membrane phospholipids in association with ROS. The process of lipid peroxidation is triggered by both enzymatic and non-enzymatic methods. Enzymatic lipid peroxidation is driven by enzymatic oxidation such as lipoxygenases (LOXs), cyclooxygenases (COXs), and Acyl-CoA synthetase long chain family member 4 (ACSL4), while non-enzymatic lipid peroxidation is driven by free radicals (Rochette et al., 2022). Among them, PUFAs can be catalysed by ACSL4 which is related to lipid metabolism, leading to excessive accumulation of lipid ROS and ferroptosis (Doll et al., 2017). Non-enzymatic lipid peroxidation driven by free radicals may be the main mechanism leading to ferroptosis in the body. Oxidative products such as hydroxyl radicals (OH•) and hydrogen peroxide (H_2_O_2_), first obtain hydrogen atoms from carbon-carbon double bond of PUFAs and react with oxygen molecules to produce lipid radicals, which obtain hydrogen atoms from other PUFAs to form new lipid peroxide radicals (LOO•) and lipid hydroperoxides (LOOH). LOO• can continuously react with PUFAs, giving the lipid peroxidation of PUFAs a cascade reaction characteristic. The degradation of PUFAs can lead to the generation of peroxidation products such as lipid peroxide (LPO) and malondialdehyde (MDA) (Abusarah et al., 2017). LOOH can further destroy the fluidity and stability of the cell membrane, increase the permeability of the cell membrane, which leads to lipid membrane rupture and ultimately lead to ferroptosis in the body (Kuang et al., 2020; Zou and Schreiber, 2020). Therefore, the enzymatic lipid peroxidation of PUFAs in membrane phospholipids catalysed by LOXs, COXs, and ACSL4 and non-enzymatic lipid peroxidation mediated by ROS are the final steps in the ferroptosis process (Figure 1).

### 2.3 Iron overload

Another characteristic of ferroptosis is iron overload. Under physiological conditions, iron, as an essential trace element, mainly participates in biological processes such as oxygen transport, adenosine triphosphate (ATP) synthesis, and immune regulation (Li et al., 2019; Kawakami and Bhullar, 2020). Simultaneously, iron, as a cofactor of iron-containing enzymes such as LOXs, EGLN prolyl hydroxylase (PHD), and cytochrome P450 oxidoreductase (POR), regulates the activities of these bonding enzymes through the redox reaction between ferrous (Fe^2+^) and ferric ions (Fe^3+^) (Zou and Schreiber, 2020). Dufrusine B et al. found through proteomic techniques that iron metabolism related proteins were dysregulated in lymphocytes of COVID-19 patients, and the expression level of 5-LOX gene was significantly increased in lymphocytes of COVID-19 patients. Iron overload in lymphocytes of COVID-19 patients upregulated the expression of 5-LOX, promoting intracellular lipid peroxidation reaction. Reminder: 5-LOX is a key target for controlling iron overload lipid peroxidation reaction (Dufrusine et al., 2022). Another study has confirmed that ALOX15 belongs to the LOX enzyme class, and its function is to promote the ferroptosis of HT1080 cells induced by small molecule compounds Erastin and RSL3, which was promoted the accumulation of lipid hydroperoxides in cell membranes. However, the specific ALOX15 inhibitor PD146176 and siRNA-mediated ALOX15 knockdown reversed the above phenomenon (Shintoku et al., 2017). Meanwhile, other studies have shown that the anti-ferroptosis biological activity of Vitamin E is determined by its metabolic active components α-Tocopherol hydroquinone, which causes the inactivation of 15-LOX activity by reducing the oxidation state of Fe^3+^ in the center of the active site of 15-LOX to the reduced state of Fe^2+^, so as to exert anti-cell lipid peroxidation reaction and prevent the occurrence of cell ferroptosis (Hinman et al., 2018). It can be seen that in the process of ferroptosis induced by iron overload, the activity of LOX was regulated through the redox reaction between Fe^2+^ and Fe^3+^, and the activity of LOX is the key molecule to inducing ferroptosis. Iron in the human body is mainly obtained by the phagocytosis of iron released from aging red blood cells by the monocyte-macrophage system, but a small amount of iron is absorbed through the duodenum and upper jejunum (Piperno et al., 2020). Iron balance is maintained primarily by the production and secretion of iron homeostasis regulators by hepatocytes, including transferrin receptor (TFR), ferritin, ferroportin (FPN), and hepcidin (Gao et al., 2019b). Among them, TFR is a transmembrane glycoprotein, which plays a role in iron uptake by binding with transferrin (TF), and transports Fe^3+^ carried by TF into the cytoplasm through endocytosis. In cells, the vast majority of Fe^3+^ is stored in ferritin, which is composed of two different subunits: ferritin heavy chain 1 (FTH1) and ferritin light chain (FTL) (Liu et al., 2021). FTH1 exhibits iron oxidase activity and is responsible for oxidising Fe^2+^ to Fe^3+^, whereas FTL contributes to the nucleation and mineralisation of iron (Chen et al., 2021b). A small portion of Fe^3+^ in the cytoplasm is reduced to Fe^2+^ under the catalysis of endosomal metal reductase, six transmembrane epithelial antigens of prostate 3 (STEAP3). Then, Fe^2+^ is transported from vesicles to the labile iron pool (LIP) in the cytoplasm by a divalent metal transporter 1 (DMT1). Most Fe^2+^ in LIP is transferred to the mitochondria to synthesise haem and iron-sulfur clusters (Fe-S clusters), and a small portion of Fe^2+^ produces a large amount of ROS through the Fenton reaction (Takadera et al., 2011; Chen et al., 2021a). However, a large amount of ROS can promote lipid peroxidation of the PUFA and oxidative phosphorylation of the mitochondria (Rochette et al., 2022). A redox equilibrium between Fe^3+^ bound by ferritin and Fe^2+^ stored exists in the LIP, through which ferritin can prevent excessive accumulation of ROS mediated by the Fenton reaction (Alkhateeb and Connor, 2013). FPN is also known as SLC40A1, it is the only known transmembrane protein mediating iron output on the mammalian cell membrane, which maintains iron homeostasis by releasing intracellular Fe^2+^ outside the cells (Liu et al., 2021). Hepcidin is a negative regulator that mainly regulates ferritin synthesis through the combination of iron regulatory protein 1 (IRP1) and iron regulatory protein 2 (IRP2) with iron response element (IRE) located in ferritin mRNA5*'*UTR. Hepcidin transports residual iron from the cell to the extracellular space through FPN to reduce intracellular iron overload (Lill et al., 2012; Gudjoncik et al., 2014). In summary, iron homeostasis regulators, such as TFR, ferritin, FPN, and hepcidin, play a key role in maintaining intracellular iron homeostasis by regulating iron uptake, storage, and efflux (Figure 1).

Under pathological conditions, iron overload increases the production of oxygen free radicals, and the accumulation of oxygen free radicals further oxidizes and damages various cell components (Rochette et al., 2015). The increase in iron uptake, iron absorption, and haem degradation could lead to iron overload (Fang et al., 2019; Gattermann et al., 2021). Studies have shown that pro-inflammatory cytokines, IL-1β and TNF-α can also lead to iron overload by inducing TFR expression (Al-Hetty et al., 2023). In addition, mitochondria are iron-rich organelles, and the destruction of their membranes leads to iron leakage in mitochondria, which substabtiallly increases the iron content in the cytoplasm and induces iron overload (Yang et al., 2022c). Other studies have found that Parkin-dependent mitophagy limits mitochondrial iron accumulation, whereas mitophagy dysfunction leads to intracellular iron overload (Li et al., 2018; Kang et al., 2019). At the same time, the destruction of the mitochondrial inner membrane further interferes with the oxidative phosphorylation process of the body, resulting in high ROS accumulation, thus promoting ferroptosis (Yang et al., 2022a). Ferritin phagocytosis mediated by nuclear receptor coactivator 4 (NCOA4) leads to autophagy degradation of ferritin and iron release, which is also an important mechanism involved in the regulation of free iron concentration in ferroptosis cells (Piperno et al., 2020). However, the occurrence of intracellular iron overload produces a large amount of ROS by continuously triggering the Fenton reaction; Consequently, large amount of ROS triggers lipid peroxidation of the cell membrane and mitochondrial membrane. This finally triggers a cascade reaction which induces ferroptosis (Valko et al., 2016; Kajarabille and Latunde-Dada, 2019; Yang et al., 2022b). Therefore, intracellular free iron is closely regulated in many ways, and iron stored in ferritin and mitochondria is very important for maintaining intracellular free iron. In addition, NCOA4-mediated ferritin phagocytosis abnormalities and mitophagy dysfunction induced *in vivo* free iron overload, which further induces ferroptosis.

## 3 Regulation of ferroptosis in osteoarthritis

### 3.1 The systemXc^−^/GSH/GPX4 regulatory axis and osteoarthritis

The systemXc^−^/GSH/GPX4 regulatory axis is crucial in the prevention of chondrocyte ferroptosis. In their study, Wang et al. (2022) examined cartilage samples obtained from the loading (L) zone and unloading (UL) zone of OA patients undergoing total knee arthroplasty (TKA). They utilized transmission electron microscopy (TEM) to observe ferroptosis-related morphological changes, such as mitochondrial membrane thickening and mitochondrial contraction, in chondrocytes from the L zone of OA cartilage. Additionally, mRNA microarray assay results demonstrated a reduction in the expression level of the ferroptosis biomarker GPX4 in chondrocytes in the L zone compared with the UL zone (Wang et al., 2022). Simultaneously, in cell experiments, it was revealed that primary murine chondrocytes were isolated and cultured under 1 MPa of mechanical stress exhibited ferroptosis-related changes in the mitochondrial structure compared with the primary murine chondrocytes cultured without mechanical stress. Real-time fluorescence quantitative PCR and Western blotting techniques were used to analyze the variations in GPX4 mRNA and protein expression levels, The results showed that mechanical stimulation led to a reduction in the expression levels of GPX4 mRNA and protein. The *in vivo* experimental results further confirmed that the GPX4 protein expression level in the cartilage tissue of the OA mouse model group was significantly reduced (Wang et al., 2022). And 10 weeks old Col2a1 CreERT GPX4^+/+^ mice were intraperitoneally injected with Tamoxifen (1 mg/d × 5 days) to obtain GPX4 conditional knockout (GPX4 CKO) mice, microscopic CT results showed that the formation of osteophytes in the knee joint of GPX4 CKO mice was significantly increased compared to WT mice, and Safranin O/Fast green staining results showed that the degree of chondrocyte damage in GPX4 CKO mice was significantly increased compared to WT mice, Immunohistochemical staining revealed a significant decrease in the expression of metabolic biomarkers aggrecan and Col2 in the OA cartilage tissue of GPX4 CKO mice, while the expression of metabolic biomarkers MMP13 and ADAMTS-5 was significantly increased (Wang et al., 2022). The above research results indicate that GPX4 is a key regulatory factor for ferroptosis of chondrocytes in the loading zone of joint cartilage and chondrocytes stimulated by high mechanical stress in OA patients. The expression level of GPX4 may have a potential connection with the progression of OA (Wang et al., 2022). Kong et al. (Kong et al., 2023) found in their study on the effect and potential mechanism of the extracellular vesicle miR-19b-3p of OA fibroblasts like synovial cells (OA FLS) on OA ferroptosis that the extracellular vesicle miR-19b-3p of OA FLS promotes cartilage ferroptosis and tissue damage by sponging SLC7A11 in OA (Kong et al., 2023). This study also found that compared with the normal control group, the expression of ferroptosis related markers SLC7A11, GSH, and GPX4 in cartilage tissue of OA patients decreased, while the expression of MDA, Fe^2+^, ACSL4, and miR-19b-3p in extracellular vesicles increased (Kong et al., 2023). Simultaneously using IL-1β Inducing chondrocytes to construct an OA chondrocyte degeneration model, the results showed a significant decrease in chondrocyte viability, with reduced expression levels of SCL7A11, GSH/GSSG, GPX4, and MMP, while increased expression levels of MDA, Fe^2+^, ASCL4, and ROS (Kong et al., 2023). The OA-FLS extracellular vesicles can further reduce the chondrocyte viability of the OA chondrocyte degeneration model, increase the downregulated expression of SCL7A11, GSH/GSSG, GPX4, and MMP in the OA chondrocyte degeneration model, and increase the upregulated expression of MDA, Fe^2+^, ASCL4, and ROS in the OA chondrocyte degeneration model (Kong et al., 2023). The intervention of ferroptosis inhibitor Fer-1 weakened the influence of OA-FLS extracellular vesicles on the above indicators. Indicating that OA-FLS extracellular vesicles promote the ferroptosis in IL-1β induced OA chondrocyte model. The author further used miR-19b-3p mimetics to overexpress miR-19b-3p, and the results showed that overexpression of miR-19b-3p reduced the chondrocyte viability of the OA chondrocyte degeneration model and reduced the expression of GPX4, GSH/GSSG, SLC7A11, and MMP in the OA chondrocyte degeneration model, but increased the expression of ACSL4, MDA, Fe^2+^, and ROS in the OA chondrocyte degeneration model (Kong et al., 2023). Inhibiting the expression of miR-19b-3p with miR-19b-3p inhibitors showed the opposite result, confirming that the miR-19b-3p in extracellular vesicles of OA FLS may be a key target leading to ferroptosis in OA. Therefore, the author predicted SLC7A11 as a miR-19b-3p target through the starBase database (starbase.sysu.edu.cn), and verified the binding of miR-19b-3p to the SLC7A11 site through luciferase reporter gene assay. The intervention of SLC7A11 overexpression plasmid showed that it can reverse the downregulation of ferroptosis related indicators GPX4, GSH/GSSG, SLC7A11, MMP and the upregulation of ACSL4, MDA, Fe^2+^, ROS by overexpression of miR-19b-3p, It is fully demonstrated that miR-19b-3p promotes ferroptosis in the OA chondrocyte degeneration model by targeting SLC7A11 (Kong et al., 2023). It can be seen that the targeted regulation of SLC7A11 in the systemXc^−^ is a key mechanism for ferroptosis in OA. In the study by He et al. (He et al., 2023) Destabilized medial meniscus (DMM) surgery was performed on the knee joint of iron overloaded mice to simulate the occurrence of knee OA. Microscopic CT examination results showed that the bone volume fraction (BV/TV), trabecular thickness (Tb. Th), and trabecular number (Tb. N) of the DMM group were significantly reduced compared to the control group, and the Osteoarthritis Research Society International (OARSI) score was significantly increased, indicating severe bone loss and cartilage tissue degeneration in the DMM group. The intervention of N-acetylcysteine (NAC) and Biochanin A (BCA) by gavage can reverse the above results (He et al., 2023). The *in vitro* experimental results further confirmed that iron overload increases the expression of ROS and lipid peroxidation in chondrocytes, reduces the systemXc^−^ (xCT), GSH, GPX4 and mitochondrial membrane potential in chondrocytes. After co-incubation with BCA, it was found that BCA can promote upregulation of the expression of xCT, GSH, GPX4 in chondrocytes, thereby resisting ferroptosis caused by lipid peroxidation damage in chondrocytes. From this, it can be seen that the traditional Chinese medicine small molecule BCA with antioxidant effects plays a role in alleviating chondrocyte ferroptosis by regulating the antioxidant systemXc^−^ (xCT)/GSH/GPX4 regulatory axis (He et al., 2023). In summary, the systemXc^−^/GSH/GPX4 regulatory axis is a key mechanism for controlling ferroptosis in OA, and its key targets may be SLC7A11 and GPX4. Among them, miRNA may target SLC7A11, but whether miRNA can target GPX4 has not been supported by relevant literature so far.

### 3.2 Lipid peroxidation and osteoarthritis

**FIGURE 2:**
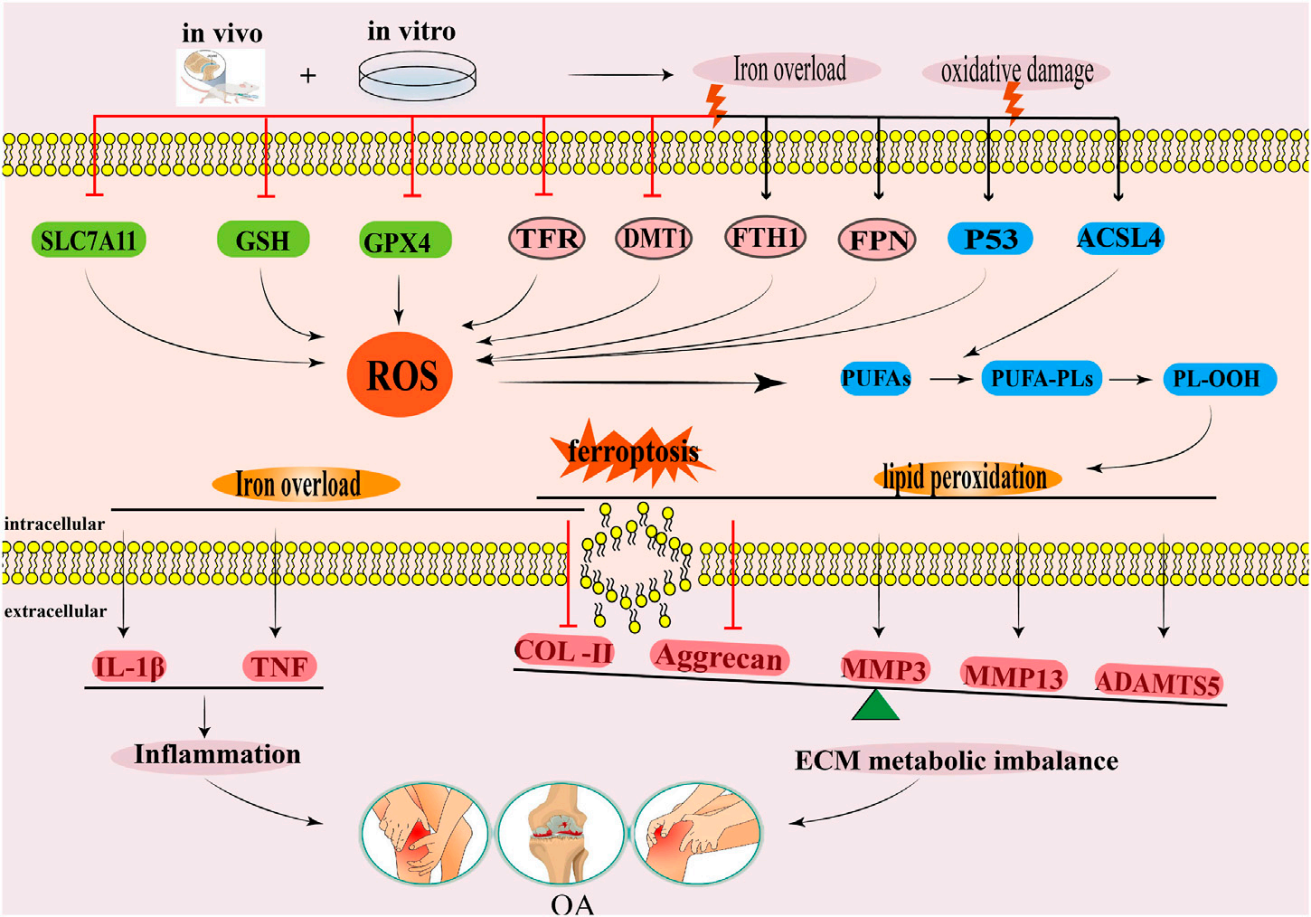
The regulatory mechanism for ferroptosis in OA. This diagram summarises the effects of iron overload and lipid peroxidation during ferroptosis on the occurrence and development of OA. The black arrow indicates activation and the red suppression arrow indicates inhibition.

When cell membrane lipids (phospholipids, glycolipids, and cholesterol) are attacked by ROS, peroxidised lipids produce lipid peroxides, such as MDA, which reacts with DNA or proteins in the body and further damages the composition and structure of the cell membrane (Foret et al., 2019; Ai et al., 2021). Gladkova (Gladkova, 2022) and Zubavlenko et al. (Zubavlenko et al., 2021) found that the levels of MDA and lipid peroxides in the serum of an anterior cruciate ligament transection (ACLT) surgery induced OA rat model increased, and the progression of OA could be alleviated by inhibiting the production of MDA and lipid peroxides. GPX4 is a lipid repair enzyme involved in liposome peroxidation, which is regulated by GSH (Ursini and Maiorino, 2020; Wei et al., 2020). Other studies have found that the levels of GPX4, GSH, and GSH/GSSG in chondrocytes exposed to lipid peroxidation in human OA lesion areas were substantially reduced, indicating that the progression of OA was accompanied by lipid peroxidation (Miao et al., 2022). Simultaneously, *in vitro* experiments that treated cells with the same concentration of tert-butyl hydrogen peroxide (TBHP) to induce oxidative damage found that the GPX4 knockdown in mouse chondroblast (ATDC5) cells and primary mouse chondroblast (MCC) cells induced a higher sensitivity in cells to oxidative stress, notably increased MDA content, and increased cell mortality. These results suggested that GPX4 knockdown enhanced the sensitivity of chondrocytes to oxidative stress by increasing their lipid peroxidation levels (Miao et al., 2022). Further *in vivo* experiments showed that, compared with the conventional control group, mice injected with specific GPX4 shRNA significantly increased osteophyte formation, size, maturity, and BV/TV of subchondral trabecular bone (STB) after ACLT surgery. The expression of anabolism biomarkers such as proteoglycans and Col2 in cartilage tissue decreased, while the expression of catabolism biomarkers MMP13, MMP3, and ADAMTS-5 increased, This indicated that downregulation of GPX4 could cause an imbalance between ECM anabolism and catabolism, which could increase OA articular cartilage degeneration and accelerate OA articular cartilage progression (Miao et al., 2022). However, whether this process involves lipid peroxidation has not been explained using *in vivo* methods. Thus, GPX4 could be a key target molecule for regulating lipid peroxidation in OA, but the specific network system through which GPX4 regulates lipid peroxidation has not been studied in detail (Figure 2).

### 3.3 Iron overload and osteoarthritis

Evidently, the iron content in the synovial fluid of an OA mouse model positively correlates with OA progression (Camacho et al., 2016). Iron overload could also lead to synovitis and hyperplasia, chondrocyte death, and the dysfunction of subchondral osteoblasts (Van Vulpen et al., 2015). Other studies have shown that iron overload destroys the iron homeostasis of chondrocytes, which leads to oxidative stress injury and apoptosis of chondrocytes (Karim et al., 2022). Therefore, the iron content of tissues must be strictly regulated (Rochette et al., 2022). Some studies have found (Burton et al., 2020) that the microCT OA scores and OARSI histology score of the whole knee joint in the guinea pig model of iron overload were higher than those in the control group, indicating that iron overload aggravated pathological injury degree of OA. Further analysis showed that iron content, and FTH-1, FPN, IL-1β, and TNF mRNA expressions in knee cartilage and the infrapatellar fat pad of guinea pigs in iron overload model groups substantially increased, while TFR, DMT1, Col2, and aggrecan mRNA expression notably decreased, indicating that the iron overload was correlated with ferritin expression, increased inflammation expression of cytokines, and inhibited cartilage tissue synthesis (Burton et al., 2020). The above results indicate that iron overload could be an important factor in the development of OA. Yao et al. revealed the relationship between iron overload in chondrocytes and OA progression for the first time (Yao et al., 2020). In the chondrocyte degeneration model induced by IL-1β and the chondrocyte iron overload model induced by ferric ammonium citrate (FAC), both chondrocyte degeneration and iron overload inhibited the expressions of GPX4 and SLC7A11 in chondrocytes, upregulated the protein expressions of P53 and ACSL4, and induced ROS accumulation in chondrocytes (Yao et al., 2020). This study also found that Ferrostatin-1 could reduce ROS levels in chondrocytes, upregulate the expressions of GPX4 and SLC7A11 in chondrocytes, and downregulate the expressions of P53 and ACSL4 in chondrocytes (Yao et al., 2020). Similarly, *in vivo* experimental results showed that a Ferrostatin-1 injection into the articular cavity increased the expressions of Col2 and GPX4 in the articular cartilage of the OA mouse model, thus reducing cartilage degradation (Yao et al., 2020). This experiment was the first to prove that inflammation and iron overload could lead to ferroptosis in OA chondrocytes. Considering that iron overload is one of the main pathological events in ferroptosis, other studies have found that (Simão et al., 2019) the knockout of the iron homeostasis regulating gene HFE model group significantly increased the iron metabolism related proteins SLC40A1, FTH1, TFR, and intracellular iron content in chondrocytes of mice, increased the expression of ROS and MMP13, and decreased the expression of Col2. These results suggested that the loss of HFE function may be another key mechanism leading to iron overload in chondrocytes (Simão et al., 2019). In summary, iron overload could induce the expressions of ROS and pro-inflammatory cytokines in chondrocytes, promote the decomposition of cartilage tissue, inhibit the synthesis of cartilage tissue, lead to articular cartilage deformation, and aggravate OA progression (Figure 2).

## 4 The regulatory role of autophagy in osteoarthritis

**FIGURE 3:**
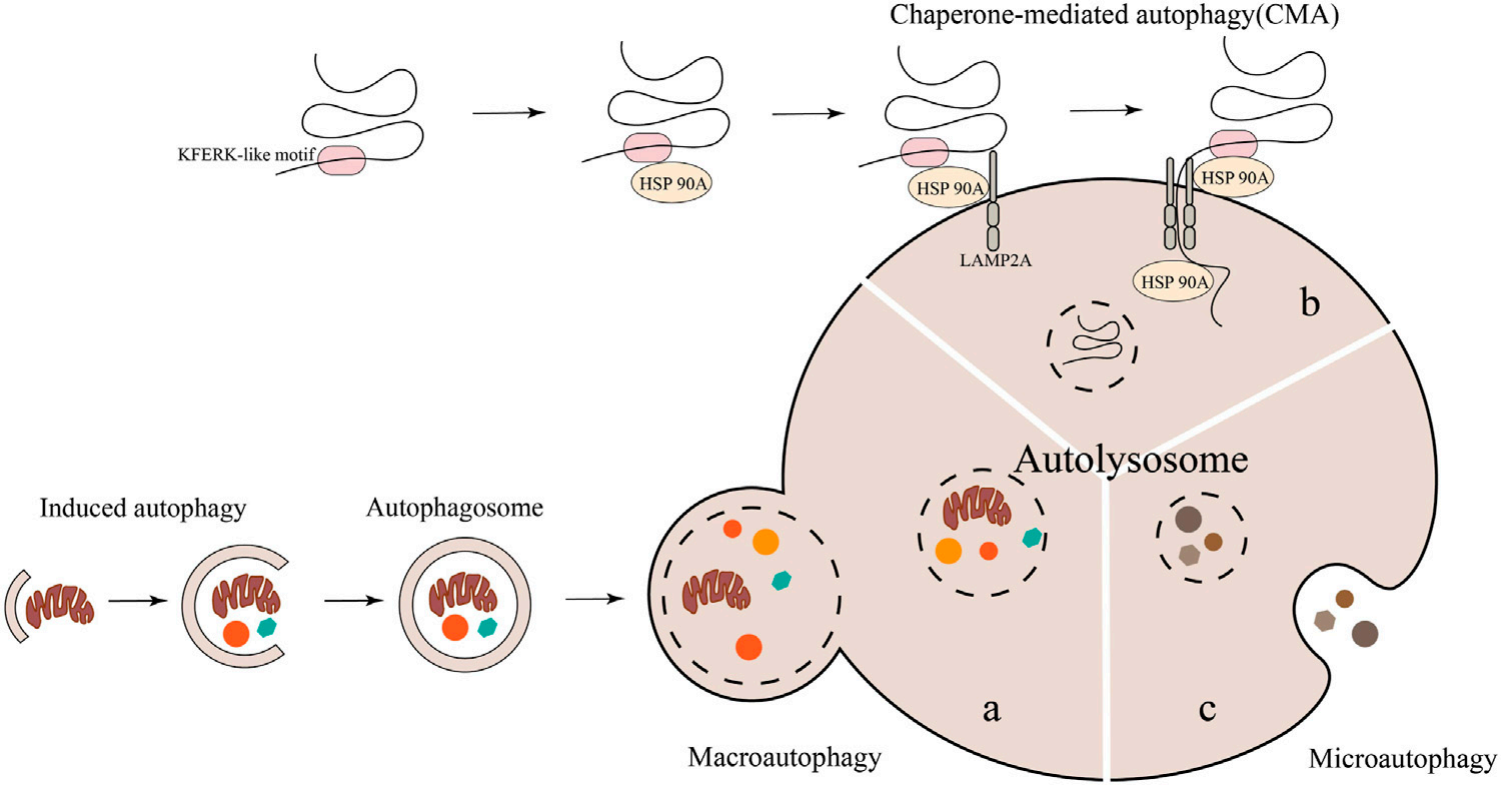
The degradation of cytoplasmic components in autophagy involves three primary types: macroautophagy, microautophagy, and molecular chaperone-mediated autophagy. Notably, macroautophagy necessitates the participation of autophagosomes.

Autophagy is a widely recognized catabolism process that is evolutionarily conserved, wherein cellular components are transported to lysosomes for degradation and subsequent recycling. In mammalian cells, autophagy is commonly categorized into three distinct types: macroautophagy or autophagy, microautophagy, and chaperone-mediated autophagy (CMA) (Wang et al., 2023). The primary focus lies on macroautophagy, commonly referred to as “autophagy,” which entails the formation of autophagosomes responsible for conveying damaged organelles, cytoplasmic proteins, and foreign microorganisms to lysosomes for degradation. Microautophagy and CMA are autophagic processes that do not necessitate the participation of autophagosomes. Instead, they rely on lysosomes/vacuoles to degrade impaired organelles, cytoplasmic proteins, and foreign microorganisms (Wang et al., 2023) (Figure 3). Previous researches have confirmed that mitophagy and CMA in macroautophagy are closely related to the occurrence and development of OA (Blanco and Rego-Pérez, 2018; Shin et al., 2019; Zeng et al., 2022a; Lorenzo-Gómez et al., 2023).

### 4.1 Macroautophagy—mitophagy

The defining feature of macroautophagy is the formation of autophagosomes, which are unique bimembrane organelles that have the ability to regulate entire organelles or degrade protein aggregates (Feng et al., 2014; Mizushima, 2018). The process of macroautophagy involves several steps: 1) Atg/ULK1 complexes play a role in initiating autophagy. 2) Various proteins such as PI3 binding protein, PI3 phosphatase, Rab protein, Atg1/ULK1 protein kinase complex, Atg9-Atg2-Atg18 complex, Vps34-Atg6/beclin1 class III PI3 kinase complex, and Atg12 and Atg8/LC3 coupling system come together to form autophagosomes (Tanida, 2011). 3) The transport and fusion of autophagosomes and lysosomes are mediated by the Double N-ethymaleiminide—sensitive factor attachment protein receptor (SNARE) protein, small GTP enzymes, and their effectors (including tethers, adaptors, and motor proteins) (Lőrincz and Juhász, 2020). 4) Hydrolase degrades the contents encapsulated in autophagosomes and releases the degradation products into the cytoplasm for the biosynthesis of biological macromolecules or participation in other metabolic pathways (Glick et al., 2010; Li et al., 2021). (Figure 3A). At present, macroautophagy is mainly divided into selective autophagy and non-selective autophagy, among which mitophagy belongs to a type of selective autophagy. Research has confirmed that mitophagy can clear damaged or dysfunctional mitochondria and maintain body homeostasis (Liu et al., 2022b). Mitophagy, a distinct form of cellular apoptosis, was initially introduced by Lemasters in 2005. This process selectively eliminates impaired or dysfunctional mitochondria, as well as surplus mitochondria, in order to uphold intracellular environmental homeostasis (Lemasters, 2005; Chen et al., 2020). Subsequent research has revealed that mitophagy can be categorized into Parkin-dependent and Parkin-independent pathways (Zhao et al., 2018).

#### 4.1.1 Parkin-dependent pathway

**FIGURE 4:**
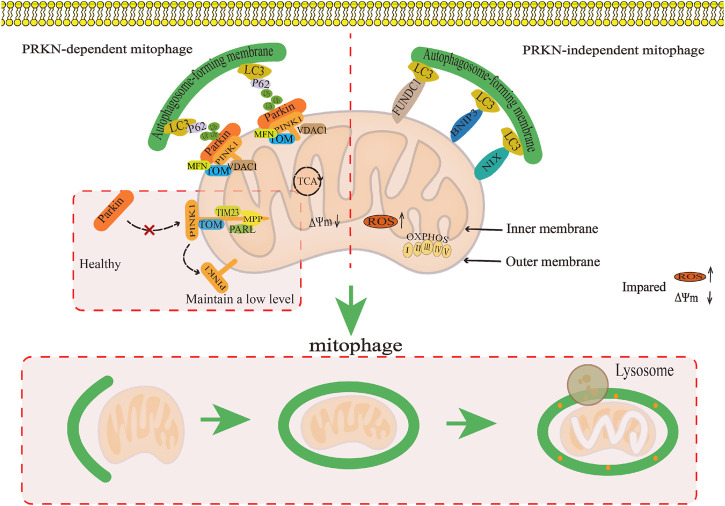
The regulatory mechanism for mitophagy. This map summarises the regulatory mechanism of the Parkin-dependent mitophagy pathway and Parkin-independent mitophagy pathway in mitophagy.

The Parkin-dependent pathway is a mitophagy pathway mediated mainly by PINK1 (PTEN induced putative kinase 1) and Parkin proteins (Clark et al., 2006). PINK1 is a kinase located in the mitochondria (Valente et al., 2004), while Parkin is an E3 ubiquitin ligase located in the cytoplasm (Shimura et al., 2000). Under physiological conditions, PINK1 is introduced into the inner mitochondrial membrane (IMM) or mitochondrial matrix via its N-terminus mitochondrial targeting sequence (MTS) through the complex formed by the translocase of the outer membrane (TOM) and translocase of the inner membrane 23 (TIM23), and then degraded into PINK1 with different molecular weights by matrix processing peptidase (MPP) and presenilin-associated rhomboid-like protein (PARL) related to the IMM rhomboid protease (Jin et al., 2010), that is, it is transferred from the mitochondria to the cytoplasm and further degraded by the proteasome in the cytoplasm, thus keeping PINK1 at a low level (Yamano and Youle, 2013). When mitochondria are damaged, PINK1 cannot be introduced into the IMM. PINK1 is anchored to the TOM of the outer mitochondrial membrane (OMM) in a voltage-dependent manner, and the E3 ubiquitin ligase Parkin is recruited from the cytoplasm to the mitochondria through its C-terminus (Yamano and Youle, 2013). Parkin recruited into mitochondria triggers ubiquitination of mitochondrial membrane proteins such as TOM, voltage-dependent anion channel 1 (VDAC1), and the mitofusin (MFN), and further recruits the protein sequestosome 1 (p62/SQSTM1) and LC3 autophagy proteins through signal cascade amplification effect. P62 binds to damaged ubiquitinated mitochondria through ubiquitin-associated (UBA) domain (Zhao et al., 2018) and is swallowed by a bimodal structure called autophagosomes, which fuse with lysosomes to degrade damaged mitochondria (Nguyen et al., 2016). It can be seen that the Parkin-dependent pathway in mitophagy pathway is mainly mediated by PINK1/Parkin proteins. It is the process of forming autophagosomes to transport damaged mitochondria to lysosomes for degradation or clearance, which is crucial for mitochondrial quality control. (Figure 4).

#### 4.1.2 Parkin-independent pathway

The Parkin-independent pathway is another mitophagy pathway that mainly dependent on non PINK1 OMM proteins. These OMM proteins include BCL2/adenovirus E1B19 kDa protein-interacting protein 3 (BNIP3), NIP3-like protein X/BNIP3-like protein (NIX/BNIP3L), containing FUN14 domain protein 1 (FUNDC1), and FK506 binding protein 8 (FKBP8) (Novak et al., 2010; Hanna et al., 2012; Liu et al., 2012; Bhujabal et al., 2017). They directly interact with the microtubule-associated protein 1 light chain 3 (MAP1LC3/LC3) through their LIR motifs, thus specifically recruiting dysfunctional mitochondria into autophagy to clear damaged mitochondria (Stolz et al., 2014; Sánchez-Martín and Komatsu, 2020). At present, the specific regulatory mechanism has not been reported in the literature (Figure 4).

#### 4.1.3 Regulation of mitophagy in osteoarthritis

**FIGURE 5:**
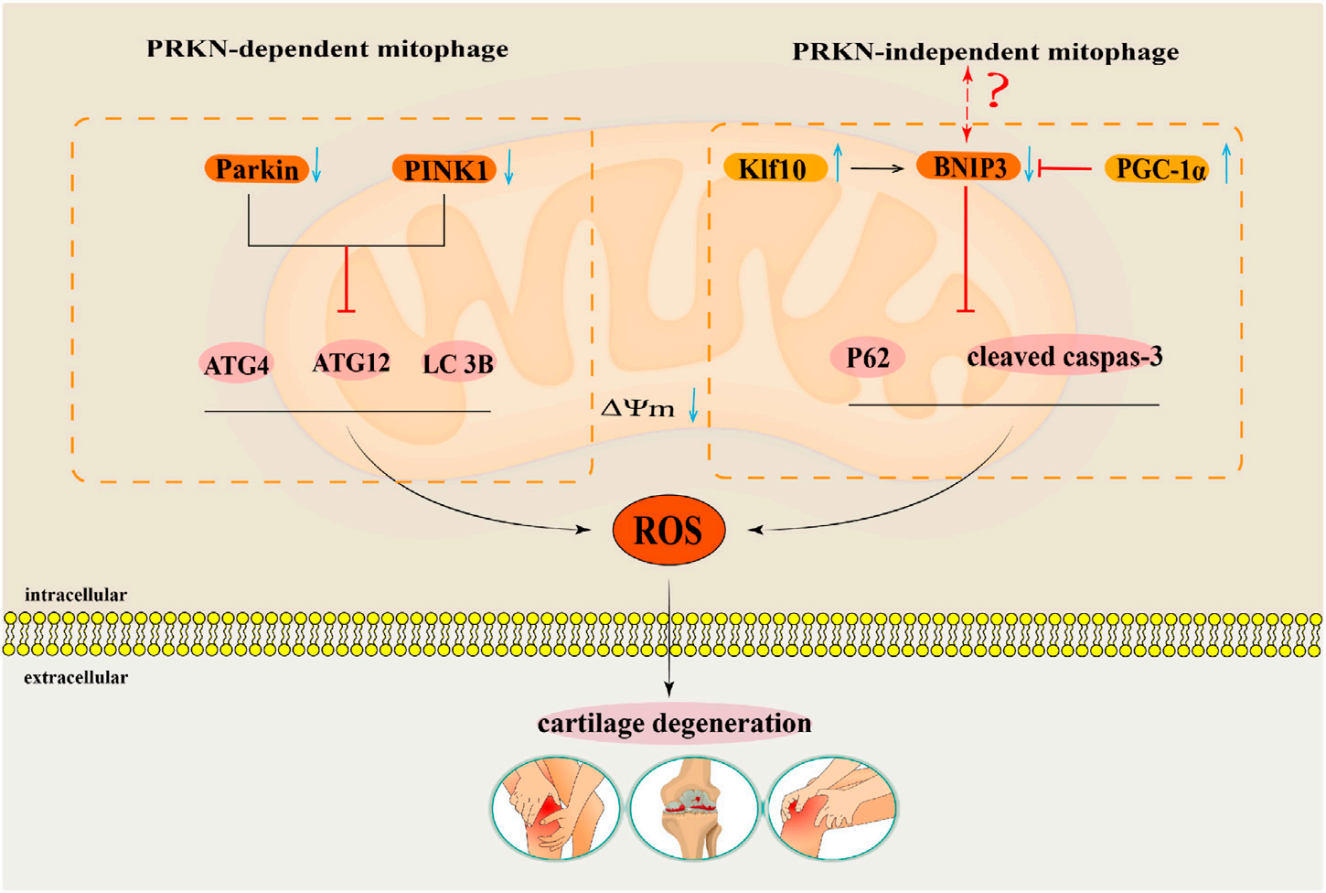
The regulatory mechanism for mitophagy in OA. This map summarises the effects of the mitophagy that Parkin-dependent and Parkin-independent pathways on the occurrence and development of OA. The black arrow indicates activation and the red suppression arrow indicates inhibition.

Reportedly, increasing evidences have indicated that enhancing mitophagy plays a key regulatory role in delaying OA progression (Blanco and Rego-Pérez, 2018; Shin et al., 2019; Zeng et al., 2022b). Notably, mitophagy function of articular chondrocytes in OA patients is weakened, which indicates that enhancing the mitophagy function may delay articular cartilage degeneration in OA patients (Rockel and Kapoor, 2017). IL-1β is related to an OA-relevant key pro-inflammatory factor, which usually simulates *in vitro* pathological features of OA conditions (Kapoor et al., 2011). Ansari et al. (2018) found that the accumulation of Parkin in chondrocytes could promote mitophagy, remove damaged and dysfunctional mitochondria in OA degenerated chondrocytes induced by IL-1β, inhibit the production of ROS, and thus prevent oxidative stress damage of OA degenerated chondrocytes induced by IL-1β. Research by Xin et al. (2022) also confirmed that upregulated Parkin eliminates damaged mitochondria in chondrocytes treated with IL-1β by enhancing mitophagy. Parkin also maintain mitochondrial membrane potential and inhibit mitochondrial apoptosis to reduce excessive accumulation of ROS, thus playing a role in delaying articular cartilage degeneration. Parkin has been suggested as the key target to regulate mitophagy in OA. Wang et al. (2020a) found that the expressions of PINK1 and Parkin in mouse chondrocytes induced by IL-1β were downregulated, the expressions of autophagy proteins Atg4, Atg12, and LC3B were downregulated, and the production of ROS was increased. The above results suggested that inhibiting the expressions of autophagy proteins in the PINK1/Parkin pathway could inhibit mitophagy of mouse chondrocytes induced by IL-1β, and increase the accumulation of ROS, thus aggravating articular cartilage degeneration. In summary, mitophagy mediated by the Parkin-dependent pathway plays a key regulatory role in cartilage degeneration in OA (Figure 5).

Tang et al. (2017) showed that the autophagy flux labeling protein p62 and apoptosis labeling protein cleaved caspase 3 were significantly upregulated in human OA chondrocytes and TBHP induced oxidative damage models of mouse OA chondrocytes. By activating BNIP3 mediated mitochondrial autophagy, the expression of p62 and cleaved caspase 3 proteins can be downregulated to counteract oxidative stress-induced apoptosis in OA mouse chondrocytes. This showed that BNIP3 mediated mitophagy played a key regulatory role in anti-apoptosis of OA chondrocytes (Tang et al., 2017). Simultaneously, Shang et al. (2023) found that Krüppel-like factors 10 (Klf10) was upregulated in aging chondrocytes, while knocking down Klf10 reduced ROS levels in aging mouse chondrocytes induced by TBHP, restored mitochondrial membrane potential, and maintained mitochondrial quality. Further studies on the mechanism showed that a downward adjustment of Klf10 resulted in over-regulation of the BNIP3-mediated mitophagy pathway, which attenuated the oxidative stress-induced aging phenotype of chondrocytes. BNIP3-mediated mitophagy has a protective effect as it delays senescence of chondrocytes subjected to oxidative stress (Shang et al., 2023). While in a study by Kim et al. (2021), mitochondrial biogenesis in OA degenerated chondrocytes induced by IL-1β was inhibited. The expression of its key regulatory factor peroxisome proliferator-activated receptor γ coactivator 1 alpha (PGC-1α)was inhibited. Knocking down PGC-1α increases the expression of BNIP3, thus activating the BNIP3-mediated Parkin-independent mitophagy pathway. The overexpression of BNIP3 induces mitochondrial depolarisation and then induces upregulation of mitophagy, which leads to an imbalance of cartilage matrix homeostasis and apoptosis of OA chondrocytes (Kim et al., 2021). It is worth noting that in the regulation of OA by BNIP3 mediated Parkin-independent mitophagy, Tang’s (Tang et al., 2017) and Shang’s (Shang et al., 2023) research results contradict with Kim’s (Kim et al., 2021) research results. Therefore, the regulatory mechanism of BNIP3 mediated Parkin-independent mitophagy pathway in the pathogenesis of OA needs further research (Figure 5).

### 4.2 Regulation of chaperone-mediated autophagy in osteoarthritis

The process known as CMA involves the delivery of a cytoplasmic protein containing a KFERK-like motif to the lysosome for degradation. This process is facilitated by the formation of a complex between a chaperone protein and lysosomal associated membrane protein type 2A (LAMP2A) (Kaushik and Cuervo, 2018) (Figure 3B). In a study conducted by Lorenzo-Gómez et al. (2023), it was demonstrated for the first time that heat shock protein 90A (HSP90A) plays a crucial role in maintaining chondrocyte homeostasis by regulating the interplay between CMA and autophagy. After studying autophagy genes in the blood of OA patients, the author found that compared with non-OA subjects, the macroautophagy related genes PTEN, MAP1LC3B, ATG4B, and GABARAP1, as well as the CMA genes HSP90AA1 and HSPA8, were significantly downregulated in the blood of OA patients. Regression analysis showed that the reduced expression level of HSP90AA1 was a risk factor for the onset of OA. The above results indicate that weakened autophagy function may be the fundamental reason for the occurrence of related pathological features in patients with knee OA (Lorenzo-Gómez et al., 2023). At the same time, the results of this study also found that the expression of HSP90A in joint tissues such as the medial cartilage, meniscus, anterior cruciate ligament (ACL), and synovial membrane of OA patients gradually decreases with the severity of OA joint tissue. The *in vivo* experimental results provide further evidence of a significant reduction in the expression level of HSP90A in both spontaneous age-related OA mouse models and surgically induced OA mouse models (Lorenzo-Gómez et al., 2023). Upon the knockout of the HSP90AA1 gene in human chondrocytes, it was observed that the absence of the HSP90AA1 gene promotes the upregulation of the aging gene p21, the cartilage degradation gene MMP13, and the expression of the inflammatory factor NF-κB/RELA and other mRNA in human chondrocytes. Additionally, increases the content of ROS and the rate of cell apoptosis, ultimately leading to oxidative stress and apoptosis in human chondrocytes. After overexpression of HSP90AA1 in human chondrocytes, it was found that the expression of autophagy regulating genes LC3II and p62 increased, while the expression of chondrocyte aging related genes p21 and p16 decreased, indicating that HSP90A activates positive regulation of autophagy. The lack of HSP90AA1 can disrupt chondrocyte homeostasis, while overexpression of HSP90A can activate the CMA gene, protecting chondrocytes from death (Lorenzo-Gómez et al., 2023). Based on the aforementioned evidence, it is evident that targeting CMA could potentially serve as an efficacious therapeutic approach in mitigating the progression of disease among individuals with OA.

## 5 Crosstalk regulation of ferroptosis and mitophagy in osteoarthritis

The pathological process of OA involves a close relationship between ferroptosis, mitophagy, and ROS metabolism. ROS may play a crucial role in regulating the crosstalk between ferroptosis and mitophagy in OA. Ferroptosis is characterized by the excessive generation of ROS, which in turn triggers lipid peroxidation and ultimately results in chondrocyte death (Miao et al., 2022). However, other studies have found that both excessive and insufficient activation of mitophagy are detrimental to the maintenance of cartilage homeostasis, and moderate activation of mitophagy can degrade damaged mitochondria in OA and reduce the production of excessive ROS (Sun et al., 2021). It is speculated that regulating mitophagy and reducing mitochondrial ROS production may inhibit ferroptosis in OA. In addition to ROS, AMPK and HIFs may also mediate OA ferroptosis and mitophagy, playing an important regulatory role in OA ferroptosis and mitophagy. However, there are currently no relevant reports on the specific crosstalk mechanism.

### 5.1 Reactive oxygen species

**FIGURE 6:**
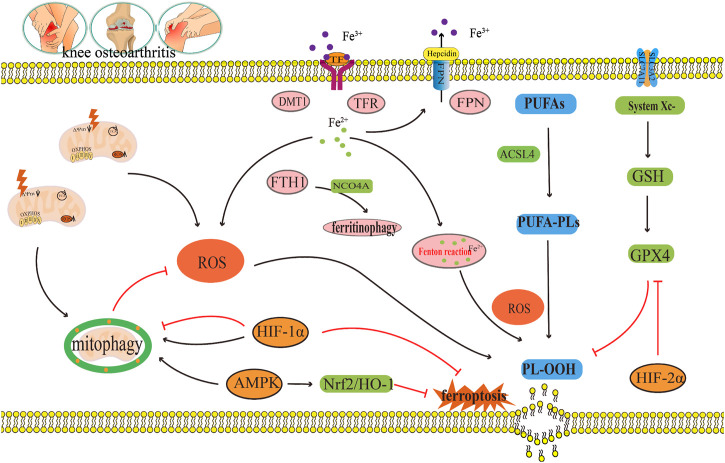
The crosstalk regulatory mechanism for ferroptosis and mitophagy in OA. In OA, iron transport related proteins TFR, FTH1, FPN, and DMT1, abnormal expression and ferritin phagocytosis mediated by NCOA4, and the imbalance of regulation and control can lead to a change in iron concentration. Iron overload produces a large amount of ROS through the Fenton reaction, which leads to lipid peroxidation damage. However, disorder in the systemXc^−^/GSH/GPX4 regulatory axis will also lead to lipid peroxidation damage, which will lead to the ferroptosis of chondrocytes. Appropriate mitophagy can alleviate ferroptosis by eliminating damaged mitochondria and reducing ROS production. This diagram briefly describes the network relationship of crosstalk regulation between ferroptosis and mitophagy from ROS, AMPK, and HIF. The black arrow indicates activation and the red arrow suppression indicates inhibition.

High intracellular ROS levels are an important inducing factor in many chronic diseases. Oxidative damage induced by ROS is known to be the main cause of senescence of OA chondrocytes (Henrotin et al., 2005). When chondrocytes were stimulated by FAC, the mitochondrial membrane potential of chondrocytes decreases substantially under iron overload, whereas the contents of ROS and lipid peroxide increase notably (He et al., 2023). Iron overload increases the production of ROS in cells, thus accelerating the progress of OA. Furthermore, erastin substantially promotes ferroptosis in chondrocytes, accumulation of ROS in erastin treated chondrocytes, and downregulates the expressions of the ferroptosis related proteins FTH1, GPX4, and SLC7A11 (Xu et al., 2022). These results indicate that ROS accumulation could be the key mechanism leading to ferroptosis in OA (Figure 6).

Mitophagy reduce the number of depolarized/damaged mitochondria, inhibit ROS production, and improve the survival rate of OA chondrocytes under pathological conditions. Jiang et al. (2022) reported that carnitine palmitoyltransferase 1A (Cpt1a) is highly expressed in aging chondrocytes and OA cartilage tissues, and that the total ROS levels in TBHP-treated chondrocytes increase substantially. The increased effect of TBHP on ROS can be reversed by knocking down Cpt1a or applying Cpt1a inhibitor (etomoxir). Simultaneously, it was found that knocking down Cpt1a decreased the expression levels of mitophagy related proteins p62 and Tomm20 and increased the ratio of LC3-II/LC3-I and the expression level of PINK1. These results suggest that Cpt1a knockdown could regulate the aging phenotype of chondrocytes through mitophagy mediated by PINK1 (Jiang et al., 2022). Liu et al. (2023) found that α-ketoglutaric acid (α-KG) can notably improve the mitochondrial respiratory function of cartilage degeneration cells induced by IL-1β, inhibit ROS production of cartilage degeneration induced by IL-1β, and enhance the expression levels of PINK1, Parkin, ATG4D, JUN, TOMM7, and DAPK2 in cartilage degeneration induced by IL-1β. These results indicate that α-KG could improve mitochondrial function and delay articular cartilage degeneration by enhancing mitophagy in OA chondrocytes, and ROS could be the key molecule of α-KG enhancing mitophagy in OA chondrocytes (Liu et al., 2023). Castro et al., 2020) also found that a decrease of mitochondrial ATP production in cartilage degeneration cells induced by IL-1β leads to reduced extracellular adenosine and adenosine A2A receptor (A2AR) expression. In addition, the downregulation of A2AR expression can further damage mitophagy, which leads to an increase in mitochondrial ROS load and mitochondrial dysfunction, thus inducing OA. In this process, the increase in ROS load caused by the damage of mitophagy could be a key molecule to the decrease in A2AR expression and the damage to OA mitochondrial function (Castro et al., 2020) (Figure 6).

In summary, ROS accumulation could lead to ferroptosis in OA, as well as, inhibit mitophagy in OA; thus, aggravating articular cartilage degeneration and accelerating the progress of OA (Figure 6). ROS is a key molecule in the regulation of mitophagy in OA and in the regulation of ferroptosis in OA (Figure 6). Indeed, ROS could be a key bridge between them.

### 5.2 Adenosine monophosphate (AMP)-activated protein kinase

Studies have shown that a decrease in AMPK activity in chondrocytes is related to OA and aging (Zheng et al., 2021). Other studies have shown that AMPK mediated by energy stress inhibits ferroptosis (Lee et al., 2020). Xie et al. (2023) cultured bone marrow mesenchymal stem cells (BMSCs) in a serum-free medium under low glucose (1.0 g L^−1^) and hypoxia (1% O_2_). The results showed that the contents of Fe^2+^, ROS, H_2_O_2_, and MDA in BMSCs increased substantially under low glucose and hypoxia, and the expression levels of ferroptosis related oxidative proteins ACSL4 and ALOX5 also increased substantially, while the expressions of ferroptosis related antioxidant proteins GPX4 and the iron storage protein FTH1 decreased notably, indicating that this culture condition could induce ferroptosis in BMSC (Xie et al., 2023). Fer-1, an ferroptosis inhibitor, can effectively reverse the ferroptosis of BMSCs induced under the above conditions. Further study on the mechanism revealed that p-AMPK and its downstream molecules PDK4 and ACC1, which regulate fatty acid synthesis, and NRF2 and GCLC, which regulate antioxidant molecules, increased substantially after Fer-1 treatment (Xie et al., 2023). In addition, the AMPK inhibitor compound C increased the number of BMSCs that died and reduced cell viability, and blocked the protective effect of Fer-1 by increasing the content of Fe^2+^, ROS, H_2_O_2_, MDA, and intracellular lipid droplets. These results suggest that AMPK plays a key regulatory role in the inhibition of ferroptosis by Fer-1 (Xie et al., 2023). Li et al. (2020) found the expression of p-AMPKα was downregulated during the occurrence and development of OA and activation of p-AMPKα could delay the articular cartilage degeneration of OA. Other studies have also found (Wan et al., 2023) that Baicalein could upregulate the protein expressions of AMPKα, AMPKβ, AMPKγ, Nrf2, and HO-1 in IL-1β-induced degenerated chondrocytes and inhibit the contents of ROS and Fe^2+^. It is speculated that baicalein can inhibit ferroptosis in IL-1β induced degenerated chondrocytes by activating the AMPK/Nrf2/HO-1 pathway (Wan et al., 2023). In summary, AMPK may be the key upstream molecule which regulates ferroptosis in OA, but, to date, the network system which facilitates AMPK regulation of ferroptosis has not been studied in detail (Figure 6).

Some studies have shown that AMPK is the central mediator of energy metabolism and a regulator of mitochondrial function (Herzig and Shaw, 2018). AMPK phosphorylation activates mitophagy (Kim et al., 2011; Laker et al., 2017). This shows that AMPK is closely related to mitophagy (Lin et al., 2020). Jin et al. (2022) found that curcumin can substantially increased the gene expressions and protein expressions of the mitophagy markers PINK1, Parkin, LC3B, P62, and Beclin1 in IL-1β-induced degenerated chondrocytes. Furthermore, it also inhibited the gene expressions and protein expressions of the inflammatory marker molecule IL-1β and the matrix degrading enzyme MMP13 (Jin et al., 2022). At the same time, curcumin can also significantly upregulate the expression of p-AMPK in IL-1β-induced degenerated chondrocytes. This result indicates that the occurrence and development of OA are accompanied by the inhibition of AMPK and mitophagy, and that AMPK may be the key molecule that activates mitophagy (Jin et al., 2022). In summary, AMPK is not only the key molecule involved in the regulation of ferroptosis in OA, but also the key molecule which regulates mitophagy in OA. In this way, AMPK may be the vital link between them (Figure 6).

### 5.3 Hypoxia-inducible factors

HIFs is the main transcriptional regulator of chondrocytes in hypoxic environments. HIFs mainly include HIF-1 and HIF-2, of which both HIF-1 and HIF-2 have α-subunit and β-subunit. Under anoxic conditions, HIF-1α and HIF-1β form heterodimers to activate various transcriptional reactions (Smythies et al., 2019). HIF-1α and HIF-2α are highly homologous in structure, but they play different regulatory roles in diseases (Gordan et al., 2007; Takeda et al., 2010). In Zhou et al.’s study, they investigated the potential role of HIF-2α in enhancing chondrocyte sensitivity to ferroptosis (Zhou et al., 2021). To examine the significance of D-mannose in inhibiting chondrocyte ferroptosis, the researchers employed transfection technology encoding small interfering RNA (si Epase1) to silence HIF-2α in chondrocytes. The results revealed that the silencing of HIF-2α significantly suppressed lipid peroxidation induced by IL-1β in chondrocytes, while simultaneously upregulating the expression of ferroptosis inhibitory genes GPX4 and SLC7A11 (Zhou et al., 2021). The overexpression of HIF-2α via adenovirus (Ad-Epas1) resulted in a decrease in chondrocyte vitality, downregulation of ferroptosis inhibitory genes GPX4 and SLC7A11, as well as lipid droplets in chondrocytes. Additionally, the expression of the oxidase gene Cpt1a increased the expression of the lipid peroxidation byproduct MDA and the ferroptosis sensitive gene Hilpda (Zhou et al., 2021). These findings suggest that the inhibition of ferroptosis by D-mannose is dependent on HIF-2α. Moreover, subsequent *in vivo* experimental validation demonstrated that the intra-articular administration of Ad-Epas1, which overexpresses HIF-2α, in the OA mouse model established following ACLT surgical resection, abrogated the beneficial impact of D-mannose on cartilage degeneration and the progression of OA in the model mice (Zhou et al., 2021). Furthermore, the intra-articular injection of Fer-1, a ferroptosis inhibitor, counteracted the influence of Ad-Epas1 on ferroptosis activity and averted cartilage impairment. Therefore, it was further determined that HIF-2α is a catabolic inducing factor that increases cartilage damage in OA, and HIF-2α has also been reported for the first time by increasing the lipid peroxidation reaction and ROS accumulation of chondrocytes, the sensitivity of chondrocytes to ferroptosis is enhanced, thereby mediating cartilage degeneration in OA (Zhou et al., 2021). Some studies have also found (Guan et al., 2023) that downregulation of HIF-1α expression could delay the progress of OA related to ferroptosis by activating the solute vector family 2 member 1 (SLC2A1), thus playing a protective role in OA. The above results suggest that the occurrence of ferroptosis in OA can be modified by regulating different subtypes of HIFs (Figure 6).

In addition, HIF-1α is closely related to mitophagy (Zeng et al., 2022a). Hu et al. (2020) found that the protein expression of HIF-1α in knee chondrocytes of patients with OA substantially increased. A hypoxic environment could also significantly upregulate the protein expression of HIF-1α in mouse knee chondrocytes, and at the same time, upregulate the expression of the mitophagy related protein p62, increase the LC3-II/I ratio, inhibit mitochondrial dysfunction, and prevent chondrocyte apoptosis (Hu et al., 2020). However, knocking down HIF-1α can reduce the expressions of the mitophagy proteins p62 and BNIP3, reduce the LC3-II/I ratio and MMP in chondrocytes, and increase apoptosis induced by hypoxia and mitochondrial ROS production. This indicates that the decrease in the HIF-1α expression level inhibits mitophagy and aggravates mitochondrial dysfunction, further leading to cartilage degeneration in OA (Hu et al., 2020). In a study by Lu et al. (2021), it was shown that a hypoxic environment can promote the increase of HIF-1α expression, increase the expression of LC3-II, and decrease the expression of P62 in degenerated chondrocytes induced by IL-1β. This indicates that an increase in expression of HIF-1α mediates an increase in autophagy to maintain chondrocyte homeostasis (Lu et al., 2021). After silencing HIF-1α, the expressions of the mitophagy proteins PINK1, Parkin, and BNIP3, and the apoptosis proteins Cleaved Caspase-3 and Caspase-3 increased. Excessive activation of mitophagy results in mitochondrial injury and apoptosis. Indicating that by suppressing HIF-1α expression can induce excessive activation of mitophagy and aggravate the progression of OA (Lu et al., 2021). In the above two studies, it was found that inhibition of HIF-1α can lead to the progression of OA. Therefore, HIF-1α is seen to have a protective effect on OA, but the difference is that the former inhibits mitophagy, while the latter promotes mitophagy. Therefore, efficient mitophagy is very important in maintaining the metabolic stability of chondrocytes. In summary, HIFs is not only the key molecule involved in the regulation of ferroptosis in OA, but it also is the key molecule involved in the regulation of mitophagy in OA. Therefore, HIFs may act as an important bridge between them (Figure 6).

## 6 Targeted therapy of osteoarthritis

### 6.1 Targeted therapy of ferroptosis in osteoarthritis

At present, the targeted therapeutic drugs for preventing and treating ferroptosis in osteoarthritis mainly include effective components of traditional Chinese medicine, iron-chelating agents, and lipophilic antioxidants, etc.

#### 6.1.1 Effective components of traditional Chinese medicine

Traditional Chinese medicine components play an important role in regulating ferroptosis. Icariin (ICA) reverses the downregulation of GPX4, SLC7A11, SLC3A2L and reduces the content of MDA and iron in synovial cells of OA model induced by lipopolysaccharide (LPS). It is suggested that ICA can inhibit LPS induced ferroptosis of OA synovial cells by activating the systemXc^−^/GPX4 regulatory axis (Luo and Zhang, 2021). Myristicin (CAD), a compound extracted from the ginger plant family, can decrease the contents of iron, ROS, and MDA in IL-1β induced degenerated chondrocytes, and increase the protein expressions of GPX4 and SLC7A11. These results suggest that CAD significantly alleviates the ferroptosis of chondrocytes induced by IL-1β. The mechanism involved may activate the SLC7A11/GPX4 signal axis to alleviate the ferroptosis of chondrocytes (Gong et al., 2023). Biochanin A (BCA), an active component of astragalus membranaceus, can reverse the upregulation of the iron transporter TFR1 and the downregulation of FPN in a mouse chondrocyte iron overload model treated with FAC to regulate the LIP content in chondrocytes (He et al., 2023). At the same time, it can upregulate the protein expression levels of systemXc^−^, GSH, and GPX4, enhance the lipid peroxidation defence system, and reduce the ROS accumulation induced by iron overload. It has been suggested that BCA can directly reduce intracellular iron concentration by inhibiting TFR1 and promoting FPN expression. In addition, the target systemXc^−^/GPX4 regulatory axis eliminates ROS accumulation and prevents lipid peroxidation, thus delaying the progression of OA (He et al., 2023). Wan et al. (Wan et al., 2023) found that baicalein, an extract of a membranaceus, can reduce the expressions of MMP13, ADAMTS5, and collagenⅠ(Col1) in human OA chondrocytes induced by IL-1β, increase the expression of Col2, and reduce the contents of lipid ROS and Fe^2+^. These results suggest that baicalein can inhibit the catabolism of the ECM induced by IL-1β and increase the anabolism of the ECM in human OA chondrocytes by inhibiting ferroptosis, thus delaying the progression of OA (Wan et al., 2023). The black tea polyphenols compound theaflavin-3,3′-digallate (TF3) can reverse the downregulated expression of FTH1, GPX4, and SLC7A11 in human OA chondrocytes induced by erastin, reduce ROS accumulation in cells, and promote Fe^2+^ storage in FTH1, thereby improving ferroptosis in OA (Xu et al., 2022). These results indicate that ferroptosis may be an important target for inducing OA, and targeting the key molecules of ferroptosis, such as GSH, GPX4, SLC7A11, ROS, FTH1, TFR1, FPN, and Fe^2+^, with effective components of traditional Chinese medicine may be an important strategy for treating OA in the future (Table 1).

**TABLE 1:** Potential drugs that can inhibit ferroptosis and/or regulate mitophagy in OA.

Bioactive substances and drugs	Composition/Type	Model/Experiment type	Effect	Molecular mechanism	References
Icariin (ICA)	Active monomers of traditional Chinese medicine	Human OA synovial cell model induced by Lipopolysaccharide (LPS)/*in vitro*	Inhibit ferroptosis	Regulating the SystemXc^−^/GPX4 regulatory axis	Luo and Zhang (2021)
Myristicin (CAD)	Ginger family extract	IL-1β Induced rat OA chondrocyte model/*in vitro*+*in vivo*	Inhibit ferroptosis	Regulating P53/SLC7A11/GPX4 regulatory axis	Gong et al. (2023)
BiochaninA (BCA)	Active ingredient of Astragalus membranaceus	FAC construction of mouse chondrocyte iron overload model+mouse DMM surgery induced OA model/*in vitro*+*in vivo*	Inhibit ferroptosis	Regulating iron levels and Nrf2/SystemXc−/GPX4 regulatory axis	He et al. (2023)
Baicalein	*Scutellaria baicalensis* extract	IL-1β Induced mouse OA chondrocyte model+mouse DMM surgical induced OA model/*in vitro*+*in vivo*	Inhibit ferroptosis	Regulating the AMPK/Nrf2/HO-1 signaling pathway	Wan et al. (2023)
Theaflavin-3,3′-digallate (TF3)	Black tea polyphenols	Human OA chondrocyte model+rat DMM surgery induced OA model/*in vitro*+*in vivo*	Inhibit ferroptosis	Regulating the Nrf2/GPX4 signaling pathway	Xu et al. (2022)
Deferoxamine (DFO)	Iron chelator	IL-1β Induced mouse OA chondrocyte model+mouse DMM surgical induced OA model/*in vitro*+*in vivo*	Inhibit ferroptosis	Regulating the Nrf2/GPX4 signaling pathway	Guo et al. (2022)
Ferrostatin-1 (Fer-1)	Lipophilic antioxidant	Human OA chondrocytes+mouse ACLT surgery induced OA model+IL-1 β And TBHP mouse OA chondrocyte model/*in vitro*+*in vivo*	Inhibit ferroptosis	GPX4 regulates ferroptosis or oxidative stress; GPX4 via MAPK/NF κ B signal pathway regulates ECM degradation	Miao et al. (2022)
Baicalin	Scutellaria baicalensis extract	IL-1β Induced rat OA chondrocytes/*in vitro*	Promoting mitophagy	Inhibiting the PI3K/AKT/mTOR pathway; Activate PINK3/Parkin and PINK1/Drp-1 pathways	He and He (2023)
Curcumin	Turmeric root and stem extract	IL-1β Induced rat OA chondrocyte model+MIA injection into bilateral knee joints to construct rat OA model/*in vitro*+*in vivo*	Promoting mitophagy	Activate the AMPK/PINK1/Parkin pathway	Jin et al. (2022)
Irisin	A soluble peptide	Mouse IL-1β Induced OA chondrocyte model+mouse DMM surgery induced OA model/*in vitro*+*in vivo*	Promoting mitophagy	Increase the expression proteins of PINK1, Park, PGC-1 α and TFAM	Wang et al. (2020b)
Zinc	Trace element	MIA treated human chondrosarcoma cells/*in vitro*	Promoting mitophagy	Upregulation of PINK1/Parkin-dependent mitochondrial autophagy	Huang et al. (2020)
UrolithinA (UA)	A metabolite of the intestinal microbiome	Human OA chondrocyte model+mouse DMM surgery induced OA model/*in vitro*+*in vivo*	Promoting mitophagy	Activating PINK1 Parkin mediated mitochondrial autophagy	D'Amico et al. (2022)
Metformin	Western medicine	IL-1β induced rat OA chondrocyte model/*in vitro*	Promoting mitophagy	Enhanced SIRT3/PINK1/Parkin signaling pathway	Wang et al. (2019)
BMSC-exosomes	Exosomes	AGEs treated primary rat chondrocyte injury model/*in vitro*	Promoting mitophagy	Increase the expression of LC3-II/LC3-I and Beclin-1 proteins	Tang et al. (2021)

#### 6.1.2 Other therapies

An iron chelating agent is a type of chelated iron that can bind iron in the body and promote iron excretion. These agents have an antioxidant role in the body by preventing the Fenton reaction. Deferoxamine (DFO) is an effective iron chelator and has been used in the treatment of iron overload-related diseases (Muñoz et al., 2011; Yao et al., 2019). Guo et al. (2022) used IL-1β to establish a chondrocyte degeneration model and established an ferroptosis model of chondrocytes induced by erastin. Transmission electron microscopy (TEM) results showed that mitochondrial damage occurred in chondrocytes in the chondrocyte degeneration model induced by IL-1β, and this damage could be reversed after treatment with the iron chelating agent DFO. At the same time, in order to further verify the effect of DFO on the ferroptosis of chondrocytes, DFO was used to treat chondrocytes treated with erastin. Results showed that DFO significantly improved the mitochondrial structural changes induced by erastin and significantly reduced the contents of MDA and Fe^2+^ in chondrocytes (Guo et al., 2022). The OA mouse model was established by a DMM surgery, and then the articular cartilage of the OA mouse model was injected with erastin. Results showed that ferroptosis occurred in the articular cartilage of the OA mouse model, however, ferroptosis in the articular cartilage of the OA mouse model could be reversed by intra-articular injection of DFO (Guo et al., 2022). The OARSI score was used as an evaluation standard of OA progression. Results showed that the OARSI score of the articular cartilage of the OA mouse model increased significantly after intra-articular injection of erastin, whereas the OARSI score of the OA mouse model decreased significantly after intra-articular injection of DFO. These results suggest that DFO can slow down the progression of OA, and the possible mechanism is to inhibit ferroptosis in OA by reducing the Fe^2+^ content in OA chondrocytes (Guo et al., 2022). As a lipophilic antioxidant, Fer-1 (Miotto et al., 2020) is a highly effective ferroptosis specific inhibitor, Its function is to eliminate oxygen free radicals produced by iron in lipid hydroperoxides. It produces an anti-ferroptosis effect similar to that of GPX4. In a study by Miao et al. (2022), Fer-1 reversed the increases in the ROS, MDA, and Fe^2+^ contents in the OA chondrocytes induced by TBHP, reversed the decreases in the GPX4, GSH, and GSH/GSSG protein expressions, and reversed the decreases in the GPX4 and FTH1 mRNA levels. These results indicate that the ferroptosis inhibitor Fer-1 can prevent TBHP induced ferroptosis in OA chondrocytes by reducing lipid peroxidation, reducing iron accumulation, and maintaining GPX4/GSH function (Miao et al., 2022). At the same time, Xu et al. (Xu et al., 2023) showed that Fer-1 can reverse the increases in Fe^3+^, ROS, and MDA, and the decrease in GSH in human OA chondrocytes induced by IL-1β. These results indicate that intra-articular injection of DFO and Fer-1 to inhibit ferroptosis in OA chondrocytes has potential as a new target or strategy for the prevention and treatment of OA (Xu et al., 2023). In summary, targeting key molecules involved in iron overload and lipid peroxidation could be an important approach to prevention and treatment of OA (Table 1).

### 6.2 Targeted therapy of mitophagy

A comprehensive and deep comprehension of targets associated with mitophagy and targeted therapy holds significant importance in the treatment of OA. The strategic focus on mitophagy and the prompt elimination of impaired mitochondria significantly contribute to the preservation of chondrocytes’ normal physiological function, thereby serving as an efficacious approach to prevent and mitigate cartilage degeneration in OA. Currently, the primary category of targeted therapeutic medications that modulate mitophagy in OA consists of active constituents derived from traditional Chinese medicine, soluble amino acids, probiotics produced by gut microbiome metabolism, trace elements zinc, metformin, and exosomes, etc.

#### 6.2.1 Effective components of traditional Chinese medicine

Traditional Chinese medicine components play an important role in regulating mitophagy. Baicalin is a natural compound from the dried root of Scutellaria baicalensis Georgi, which has been found to have many pharmacological effects such as anti-inflammatory and anti-tumour, and can also improve immune regulation (Jiang et al., 2020). Baicalin (He and He, 2023) was shown to upregulate the expression levels of Bcl-1, Beclin3, LC3-II/LC1-I, p-Drp1, PINK1, and Parkin in rat chondrocytes induced by IL-1β, promote cell viability, autophagy, and mitophagy of rat chondrocytes induced by IL-1β, and increase MMP. Thus, it can inhibit apoptosis of rat chondrocytes induced by IL-1β. These results suggest that baicalin may activate mitophagy induced by IL-1β through the PINK1/Parkin pathway, thus alleviating chondrocyte injury (He and He, 2023). Curcumin (Jin et al., 2022) is a polyphenol compound extracted from the turmeric rhizome, which can significantly increase the gene expressions and protein expressions of the mitophagy markers PINK1, Parkin, LC3B, P62, and Beclin1 in degenerated chondrocytes induced by IL-1β. It can also inhibit the gene expressions and protein expressions of the inflammatory markers MMP13 and IL-1β. This indicates that curcumin can delay cartilage degeneration by activating mitophagy in chondrocytes (Jin et al., 2022). These studies confirm that mitophagy targeted by effective components in traditional Chinese medicine may be another hot topic in OA treatment in the future (Table 1).

#### 6.2.2 Others therapies

Wang et al. (2020c) discovered that irisin is a soluble peptide containing 112 amino acids, which regulates bone turnover and muscle homeostasis. Irisin can increase the losses of PINK1 and Parkin expressions induced by IL-1β in mouse chondrocytes to enhance mitophagy, thus reducing mitochondrial hyperdivision and increasing mitochondrial fusion, and delaying the progression of OA. This indicates that the Irisin recombinant protein enhances mitophagy in mouse chondrocytes induced by IL-1β to regulate mitochondrial dynamics, thereby achieving the goal of preventing OA progression (Wang et al., 2020a). Huang et al. (2020) studied the protective effect of zinc on human chondrosarcoma SW1353 cells treated with monosodium iodoacetate (MIA). They found that zinc can resist cartilage damage caused by MIA treatment by reversing low levels of ATP and upregulating the Parkin-dependent mitophagy pathway, indicating its potential protective effect on OA. D’Amico et al. (D'Amico et al., 2022) found that urolithin A (UA), a metabolite of the intestinal microbiome, inhibits chondrocyte degeneration by activating the PINK1/Parkin mediated pathway in OA patients to enhance their mitophagy and mitochondrial respiration, thus exerting a curative effect. In addition, some studies (Wang et al., 2019) have found that metformin can enhance mitophagy by upregulating the expressions of SIRT3, the autophagy-related proteins PINK1 and Parkin, and the ratio of LC3II/LC3I in mouse OA degenerated chondrocytes stimulated by IL-1β. Metformin can also cause the downregulation of the expressions of the MMP3 and MMP13, and the upregulation of the expression of Col2 to alleviate ECM catabolism in mouse OA degenerated chondrocytes induced by IL-1β. However, 3-TYP (SIRT3 inhibitor) can reverse the above results, suggesting that SIRT3 may be the upstream target molecule of PINK1/Parkin in the mitophagy pathway. Metformin can offset the imbalance of anabolism and catabolism in OA degenerated chondrocytes induced by IL-1β by activating the PINK1/Parkin-dependent mitophagy pathway through SIRT3 (Wang et al., 2019). Therefore, the Parkin-dependent mitophagy pathway plays an important role in preventing the progression of OA (Table 1).

Mesenchymal stem cell exosomes (MSC-exosomes) play an important role in cartilage repair by promoting the proliferation and invasion of chondrocytes in OA rats and inhibiting apoptosis (Zhang et al., 2018). Tang et al. (2021) treated primary rat chondrocytes with advanced glycation end products (AGEs) to induce cell injury and found that BMSC exosomes enhanced the expressions of the autophagy-related proteins LC3-II/LC3-I and Beclin-1 and enhanced mitophagy in AGE-treated chondrocytes. Drp1 overexpression promotes apoptosis by inhibiting the expressions of the autophagy-related proteins LC3-II/LC3-I and Beclin-1. BMSC exosomes can reverse mitophagy inhibited by the upregulation of Drp1 expression. Therefore, BMSC exosomes inhibit chondrocyte apoptosis by regulating Drp1-mediated mitophagy, which may be an effective target for OA treatment (Tang et al., 2021). These results indicate that enhancing mitophagy in OA chondrocytes may become a new strategy for OA prevention and treatment and may also become an effective target for screening OA prevention and treatment drugs in the future (Table 1).

## 7 Summary and prospects

**FIGURE 7:**
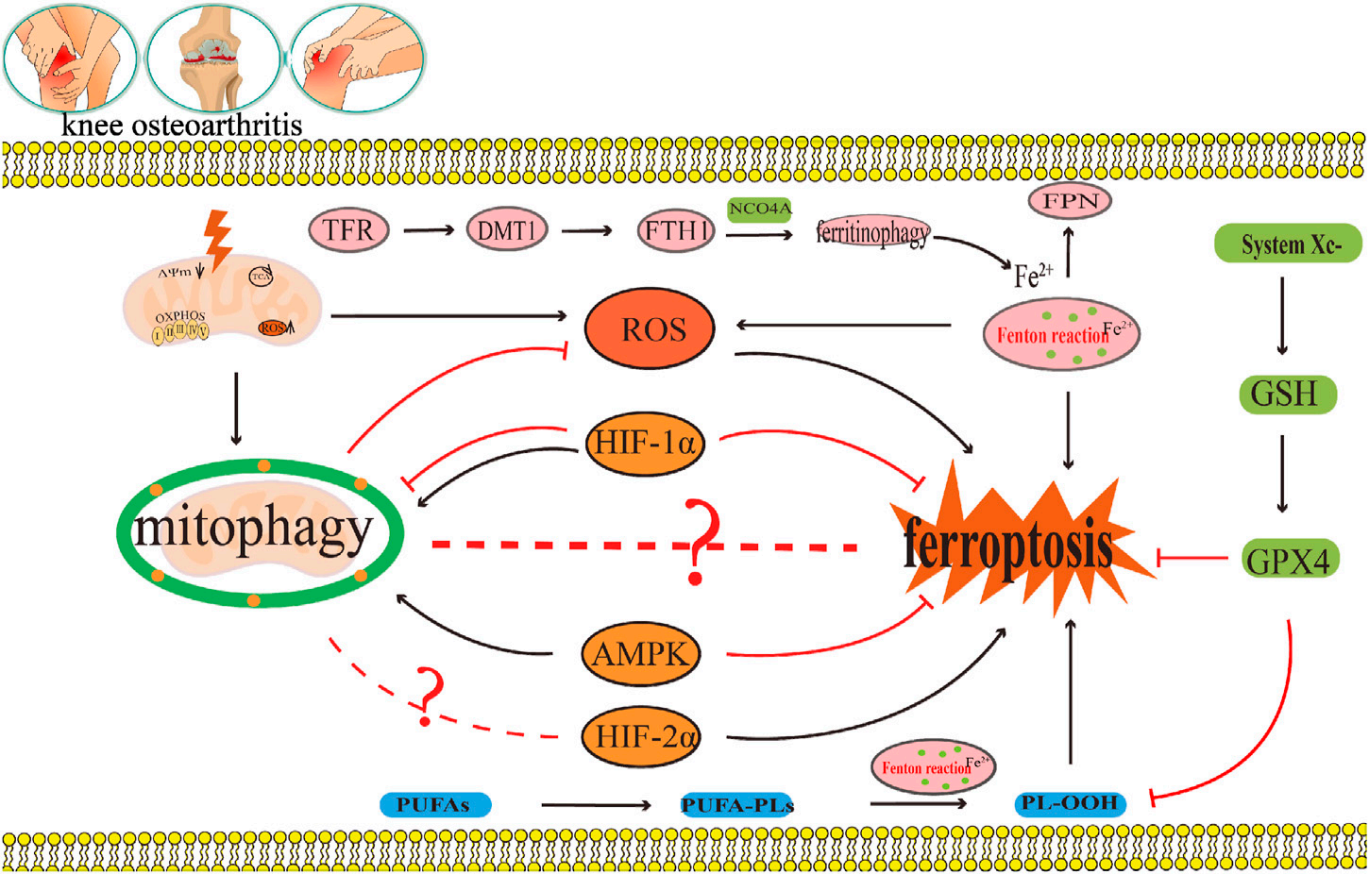
OA can delay ferroptosis by eliminating damaged mitochondria and reducing ROS production. This area will receive much research interest in the future as researchers continue to explore the target molecules of crosstalk that regulate OA ferroptosis and mitophagy, and the crosstalk regulatory mechanism. The black arrow represents activation and the red suppression arrow represents inhibition.

During the occurrence and development of OA, disorders in iron uptake, storage, and excretion, as well as abnormal expression of iron transport related proteins TFR, FTH1, FPN, and DMT1, as well as imbalance in the regulation of NCOA4 mediated ferritin phagocytosis, can lead to changes in iron concentration. Iron overload generates a large amount of ROS through the Fenton reaction, leading to lipid peroxidation damage (Figure 7). The dysfunction of the systemXc^−^/GSH/GPX4 regulatory axis can also lead to lipid peroxidation damage, leading to chondrocyte ferroptosis (Figure 7). On the other hand, both excessive and insufficient activation of mitophagy are detrimental to cartilage homeostasis. The moderate activation of mitophagy can alleviate OA by eliminating damaged mitochondria and reducing the production of mitochondrial ROS, as well as the release of mitochondrial ferritin free iron to alleviate oxidative stress response. Chaperone proteins such as HSP90A mediated autophagy can reduce the production of ROS in chondrocytes, which is a key mechanism for maintaining chondrocyte homeostasis. ROS accumulation can not only lead to the occurrence of ferroptosis in OA, but also inhibit mitophagy and CMA in OA, thereby exacerbating articular cartilage degeneration and accelerating the progression of OA. ROS is not only a key molecule regulating mitophagy and CMA in OA, but also a key molecule regulating ferroptosis in OA. ROS may be their key bridge. However, there is currently no relevant research report on whether ROS crosstalk regulates OA microautophagy or crosstalk regulates OA microautophagy and ferroptosis both domestically and internationally. In addition to ROS, this review also found that AMPK and HIFs play important regulatory roles in mediating OA ferroptosis and mitophagy. There is currently no relevant report on whether AMPK and HIFs can mediate OA ferroptosis and other forms of autophagy (microautophagy, CMA). In addition, this review also found that the effective ingredients and other substances of traditional Chinese medicine can also exert therapeutic effects by targeting OA ferroptosis and mitophagy. However, the specific network mechanism of crosstalk regulating OA ferroptosis and mitophagy has not been thoroughly studied. Furthermore, apart from ROS, AMPK, and HIFs, there exist additional molecules and distinct network regulatory mechanisms that facilitate the crosstalk regulation of OA ferroptosis and mitophagy, as well as other forms of autophagy such as microautophagy and CMA, However, there is currently a lack of relevant literature on this topic. Consequently, investigating target molecules involved in the regulation of crosstalk between OA ferroptosis and mitophagy, as well as other forms of autophagy (microautophagy, CMA), and elucidating their underlying regulatory mechanisms will emerge as a prominent area of research in the future.
